# Safety and Feasibility of Combining On-Demand Selective Locoregional Treatment with First-Line Atezolizumab Plus Bevacizumab for Patients with Unresectable Hepatocellular Carcinoma

**DOI:** 10.3390/curroncol31030117

**Published:** 2024-03-15

**Authors:** Tasuku Nakabori, Sena Higashi, Yutaro Abe, Kaori Mukai, Toshiki Ikawa, Koji Konishi, Noboru Maeda, Katsuyuki Nakanishi, Shinichiro Hasegawa, Hiroshi Wada, Kazuyoshi Ohkawa

**Affiliations:** 1 Department of Hepatobiliary and Pancreatic Oncology, Osaka International Cancer Institute, Osaka 541-8567, Japan; 2 Department of Radiation Oncology, Osaka International Cancer Institute, Osaka 541-8567, Japan; 3 Department of Diagnostic and Interventional Radiology, Osaka International Cancer Institute, Osaka 541-8567, Japan; 4 Department of Gastroenterological Surgery, Osaka International Cancer Institute, Osaka 541-8567, Japan

**Keywords:** hepatocellular carcinoma, atezolizumab plus bevacizumab, locoregional treatment, combination therapy, surgical resection, transarterial chemoembolization, radiofrequency ablation, stereotactic body radiotherapy

## Abstract

Various locoregional treatments for localized hepatocellular carcinoma (HCC) have been developed. This retrospective study investigated the safety and feasibility of combining on-demand selective locoregional treatment for residual lesions after tumor shrinkage (complete response [CR] oriented) or for solitary or few drug-resistant lesions (progressive disease (PD) salvage) with first-line atezolizumab plus bevacizumab (atezo/bev) for unresectable HCC. Twenty-nine patients with unresectable HCC were included. Fourteen locoregional treatments were performed (CR oriented, 7; PD salvage, 7) in ten patients in the combination-therapy group. All patients in the combination-therapy group successfully achieved a CR or PD salvage status after the planned locoregional treatment. The objective response rate of the combination-therapy group (80.0%) was higher than that of the atezo/bev alone group (21.1%; *p* = 0.005). Progression-free survival (PFS) and overall survival (OS) were longer in the combination group (medians for PFS and OS not reached) than in the atezo/bev alone group (median PFS, 7.4 months; median OS, 19.8 months) (PFS, *p* = 0.004; OS, *p* < 0.001). The albumin–bilirubin score did not change, and no severe complications occurred after locoregional treatment. When performed in a minimally invasive manner, on-demand selective locoregional treatment combined with first-line atezo/bev could be safe and feasible for unresectable HCC.

## 1. Introduction

Hepatocellular carcinoma (HCC) is the sixth most common cancer and the fourth leading cause of cancer-related deaths worldwide [1]. Potentially curative treatments, such as resection or ablation, are adopted for patients with Barcelona Clinic Liver Cancer (BCLC) early-stage HCC. For patients with BCLC early-stage HCC with three or fewer lesions, each measuring ≤ 3 cm, radiofrequency ablation (RFA) is considered to have the same therapeutic effect as surgical resection, with the advantage of being less invasive [2]. Transarterial chemoembolization (TACE) is suitable for local control for some patients with BCLC intermediate-stage HCC. Systemic therapy has been indicated for patients with advanced BCLC disease and has recently preceded TACE for intermediate-stage HCC, particularly for HCCs beyond the up-to-seven criteria [3,4].

Systemic chemotherapy with molecular-targeted agents, such as sorafenib [5] and lenvatinib [6], has been developed and clinically implemented since the late 2000s. Recently, the use of atezolizumab plus bevacizumab, a combination of immune checkpoint inhibitors and vascular endothelial growth factor antibodies, has significantly improved the prognosis of unresectable HCC compared to sorafenib [7,8]; therefore, an atezolizumab plus bevacizumab combinatorial treatment has been approved as a first-line systemic chemotherapy for unresectable HCC worldwide [3,9,10,11]. However, only 30% of patients who receive atezolizumab plus bevacizumab achieve a satisfactory response. The complete response (CR) rate is only 8%, while the overall survival (OS) and progression-free survival (PFS) are suboptimal at 19.2 months and 6.9 months, respectively [7,8]. Therefore, improved therapeutic strategies for systemic chemotherapy are required to overcome the poor prognosis of unresectable HCC.

Locoregional treatments like RFA can induce immunogenic cell death and are thought to enhance the immune response by releasing tumor antigens or modulating the tumor microenvironment [12,13,14]. Consequently, combining locoregional treatments with immunotherapy is expected to have a synergistic effect. Therapeutic strategies for local control comprising the addition of on-demand locoregional treatment to systemic chemotherapy, that is, curative locoregional treatment after tumor reduction by systemic chemotherapy or locoregional treatment for focal drug-resistant lesions, may prolong the administration period or improve clinical outcomes. Curative locoregional treatment after tumor reduction using systemic chemotherapy has been proposed [15]. However, the clinical significance of sequential systemic-selective locoregional therapy for unresectable HCC remains unclear. We analyzed the safety and feasibility of combining on-demand selective locoregional treatment, such as surgical resection, RFA, TACE, or stereotactic body radiotherapy (SBRT), with first-line atezolizumab plus bevacizumab for patients with unresectable HCC.

## 2. Materials and Methods

### 2.1. Study Population

We retrospectively collected clinical data of patients with unresectable HCC who received atezolizumab plus bevacizumab as the first-line systemic chemotherapy at the Osaka International Cancer Institute, Osaka, Japan. HCC was confirmed according to the diagnostic criteria of the American Association for the Study of Liver Diseases based on histological or radiological findings using dynamic contrast-enhanced computed tomography (CT) or gadolinium-ethoxybenzyl diethylenetriamine pentaacetic acid-enhanced magnetic resonance imaging (EOB-MRI) [16,17]. The HCC stage was determined according to the BCLC staging system [18,19]. The inclusion criteria were as follows: (1) initiation of atezolizumab plus bevacizumab treatment between November 2020 and September 2022 and (2) an Eastern Cooperative Oncology Group performance status score between zero and one. The exclusion criteria were as follows: (1) an observation period of less than 8 weeks; (2) lack of contrast-enhanced images of the liver; and (3) the presence of other advanced cancers as comorbidities. The initiation date of atezolizumab plus bevacizumab was determined at the start of the follow-up. The follow-up period concluded on 31 January 2024. The median observation time was 750 days (range, 60–1192 days). The study was performed in accordance with the Declaration of Helsinki and was approved by the Institutional Review Board for Clinical Research at the Osaka International Cancer Institute (approval number 22183), which waived the requirement for informed consent. The opt-out method was provided to the patients on our hospital’s website.

### 2.2. Atezolizumab Plus Bevacizumab Treatment

Patients received 1200 mg atezolizumab plus 15 mg/kg bevacizumab every three weeks. If an unacceptable adverse event related to atezolizumab plus bevacizumab occurred, then the use of either one drug or both drugs was interrupted until the symptoms resolved to grade 1 or less according to the National Cancer Institute Common Terminology Criteria for Adverse Events (version 5.0) based on the manufacturer’s guidelines for atezolizumab plus bevacizumab. Atezolizumab plus bevacizumab administration was discontinued at 21 days, 21 days, 7 days, or 42 days prior to RFA, TACE, radiotherapy, or surgical resection, respectively. After these treatments, atezolizumab plus bevacizumab administration was resumed or withdrawn, depending on the individual case.

### 2.3. RFA, TACE, and Radiotherapy Administration

RFA was performed percutaneously for ≤3 cm and four or fewer intrahepatic lesions under ultrasound guidance, or for one lung metastasis of ≤2 cm under CT guidance. A 480 kHz generator and an internally cooled radiofrequency electrode (Arfa RF ablation system; Japan Lifeline, Tokyo, Japan; or VIVA RF system; STAR Med, Gyeonggi, Korea; or Cool-tip radiofrequency system; Covidien, Boulder, CO, USA) were used based on the operator’s decision. One day after RFA, contrast-enhanced CT was performed to evaluate the therapeutic effects and complications.

TACE was performed for a single intrahepatic lesion measuring ≤ 5 cm by injecting poppy-seed oil (Lipiodol; Guerbet Japan, Tokyo, Japan) plus epirubicin (Nippon Kayaku Co. Ltd., Tokyo, Japan), followed by an injection of porous gelatin particles (Gelpart; Nippon Kayaku Co. Ltd., Tokyo, Japan), as previously reported [20].

For SBRT, therapeutic doses were delivered as follows: 40 Gy in ten fractions for ≤5 cm and two or fewer bone metastases, or 50 Gy in ten fractions for inferior vena cava tumor thrombosis, including the responsible intrahepatic lesion. Treatments were delivered using a linear accelerator, as previously reported [21].

### 2.4. HCC Treatment and Decision Regarding Combination Therapy

At a weekly multidisciplinary conference, the optimal treatment for each patient, including the most suitable locoregional treatment, was determined by hepatologists, surgeons, oncologists, and radiologists. Locoregional treatment was combined with atezolizumab plus bevacizumab for two purposes: first, to reduce or eliminate the remaining viable lesion after tumor shrinkage by atezolizumab plus bevacizumab to achieve CR (CR-oriented therapy) and, second, to treat solitary or few drug-resistant lesions (progressive disease [PD] salvage therapy). Drug resistance was defined as the growth of existing lesions or the appearance of a new tumor during atezolizumab plus bevacizumab treatment (Supplementary Figure S1). All patients with residual tumors or drug-resistant lesions that met the criteria mentioned above in Section 2.3 received locoregional treatment, which was repeated if deemed suitable.

### 2.5. Assessment of Therapeutic Responses, Hepatic Reserve, and Complications

Therapeutic responses were assessed using dynamic enhanced CT or EOB-MRI according to the Response Evaluation Criteria in Solid Tumors (RECIST) version 1.1 [22] as a CR, partial response (PR), or stable disease (SD). In determining PD, the RECIST criteria were modified such that neither tumor growth nor the appearance of a new tumor was regarded as PD if the tumor could be radically cured with locoregional treatment, as adopted in previous studies of locoregional treatment combined with molecular-targeted agents [23,24]. Cases of intensive tumor growth that could not be controlled by locoregional treatment, the appearance of multiple new lesions, or metastases to other organs were regarded as PD. Dynamic enhanced CT or EOB-MRI was performed every six to nine weeks during atezolizumab plus bevacizumab treatment. CT or MRI was additionally performed as required, for example, after surgical resection, RFA, or TACE. Objective response rates (ORRs) were defined as the sum of the CR and PR rates, and disease-control rates were defined as the sum of the CR, PR, and SD rates. PFS was calculated from the date of initiation of atezolizumab plus bevacizumab until our defined PD or death date. OS was calculated from the date of initiation of atezolizumab plus bevacizumab treatment until the date of death or the last follow-up examination.

Child–Pugh scores, albumin–bilirubin (ALBI) scores, and modified ALBI grades were used to assess the hepatic reserve [25,26]. The severity of adverse events was retrospectively graded according to the National Cancer Institute Common Terminology Criteria for Adverse Events (CTCAE; version 5.0). For liver injury, the severity was defined as the highest CTCAE grade of either alanine aminotransferase (ALT) or total bilirubin, as previously reported [27]. The Clavien–Dindo classification [28] system was adopted to assess locoregional treatment-related complications, as previously reported [23].

### 2.6. Statistical Analysis

Continuous variables were expressed as medians (ranges) and were compared using the Mann–Whitney U test or Wilcoxon signed-rank test, as appropriate. Categorical variables were expressed as numbers and were compared using Pearson’s chi-square test or Fisher’s exact test, as appropriate. Analyses of PFSs and OSs were performed using the Kaplan–Meier method, and comparisons were conducted using the log-rank test. PFSs, OSs, hazard ratios, and 95% confidence intervals (CIs) were determined by multivariate Cox proportional hazards modeling. Variables with a *p*-value <0.1 in the univariate analysis were included in the multivariate analysis. Differences were considered statistically significant at *p* < 0.05. All statistical analyses were performed using ‘EZR’ (Saitama Medical Center, Jichi Medical University, Saitama, Japan), which is a graphical interface for the R commander software package for Windows (version 1.61; R Foundation for Statistical Computing, Vienna, Austria) [29].

## 3. Results

### 3.1. Patient Characteristics

A total of 29 patients were enrolled in this study. The baseline characteristics of all patients at the time of atezolizumab plus bevacizumab initiation are shown in Table 1. Ten patients underwent locoregional treatment, such as surgical resection, RFA, TACE, or SBRT with atezolizumab plus bevacizumab treatment (combination-therapy group), and 19 patients underwent systemic chemotherapy alone with atezolizumab plus bevacizumab treatment (atezolizumab plus bevacizumab alone group). Representative cases of sequential atezolizumab plus bevacizumab with locoregional CR-oriented treatment and sequential atezolizumab plus bevacizumab with locoregional PD salvage treatment are shown in Supplementary Figures S2 and S3, respectively. The baseline characteristics of patients who received locoregional treatment in addition to atezolizumab plus bevacizumab (combination-therapy group) and the atezolizumab plus bevacizumab alone group, recorded at the initiation of systematic therapy, are shown in Table 1. At the initiation of atezolizumab plus bevacizumab, there were no significant differences in age, sex, etiology, hepatic reserve, or tumor-related factors between the two groups. All patients with BCLC Stage B had HCC beyond the up-to-seven criteria (substage B2) [19].

### 3.2. Therapeutic Responses, PFS, and OS

Among the ten patients in the combination-therapy group, 14 locoregional treatments (RFA, *n* = 9; SBRT, *n* = 2; surgical resection, *n* = 2; and TACE, *n* = 1) were performed. A summary of the treatment information for the combination group is shown in Supplementary Table S1. Seven treatments were CR-oriented, while the remaining seven were PD salvage. The median duration from the initiation of atezolizumab plus bevacizumab to the first locoregional treatment was 224 days (range, 69–426 days). Nine patients resumed atezolizumab plus bevacizumab after ten locoregional treatments. The median discontinuation period of atezolizumab plus bevacizumab before and after locoregional treatment was 49 days (range, 28–77 days). Tumors other than the lesions targeted by locoregional treatment did not progress, and no new lesions appeared during the discontinuation period of atezolizumab plus bevacizumab. The optimal overall response, ORRs, and disease-control rates are listed in Table 2. The ORR of the combination-therapy group (80.0%) was higher than that of the atezolizumab plus bevacizumab alone group (21.1%) for the entire study period (*p* = 0.005).

**Figure 1:**
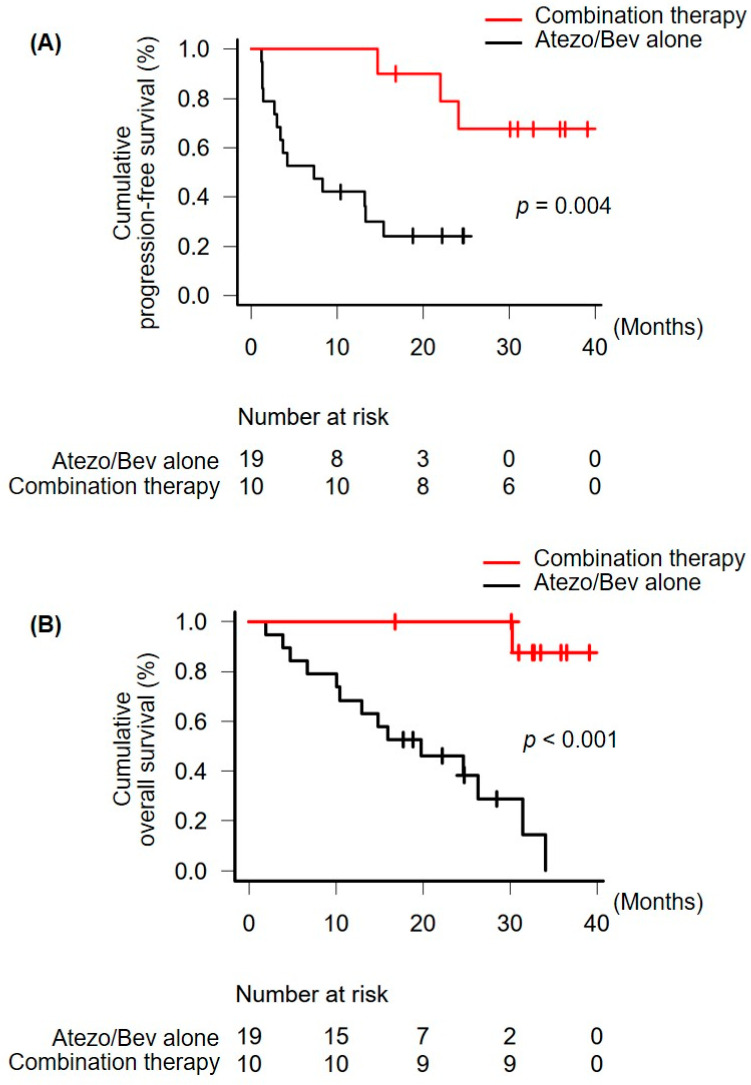
Comparisons of the combination-therapy and atezolizumab plus bevacizumab alone groups in terms of (**A**) PFS and (**B**) OS. Atezo/Bev, atezolizumab plus bevacizumab; PFS, progression-free survival; OS, overall survival.

During the observation period, three patients in the combination-therapy group and 14 patients in the atezolizumab plus bevacizumab alone group were classified as PD. One patient in the combination-therapy group and 14 patients in the atezolizumab plus bevacizumab alone group died during the study period. The PFS of the combination-therapy group (median, not available; 95% CI, 14.7 months—not available) was longer than that of the atezolizumab plus bevacizumab alone group (median, 7.4 months; 95% CI, 2.8–15.4 months; *p* = 0.004) (Figure 1A). The OS of the combination-therapy group (median, not available; 95% CI, 30.3 months—not available) was longer than that of the atezolizumab plus bevacizumab alone group (median, 19.8 months; 95% CI, 10.1–31.5 months; *p* < 0.001) (Figure 1B). Factors independently related to PFS and OS were analyzed. Regarding PFS, variables with a *p*-value < 0.10 in the univariate analysis, namely a baseline serum des-γ-carboxy prothrombin (DCP) level ≥ 1000 mAU/mL, and performance of the locoregional treatment, were included in the multivariate logistic regression model (Table 3). Locoregional treatment was independently associated with PFS (*p* = 0.004). On the other hand, regarding OS, variables with a *p*-value < 0.10 in the univariate analysis, including the ALBI grade, a baseline serum DCP level ≥ 1000 mAU/mL, and performance of locoregional treatment, were included in the multivariate logistic regression model (Table 4). Locoregional treatment was also independently associated with OS (*p* = 0.003). Five patients (50.0%) in the combination-therapy group achieved CR. In contrast, in the atezolizumab plus bevacizumab alone group, two patients (10.5%) achieved CR.

### 3.3. Adverse Events, Locoregional Treatment-Associated Complications, and Changes in the Hepatic Reserve after Locoregional Treatment

To evaluate the safety of combining locoregional treatment with atezolizumab plus bevacizumab, the total adverse events experienced during the atezolizumab plus bevacizumab treatment were analyzed (Table 5). The frequency of adverse events of any grade, as well as grade ≥ 3 adverse events, did not significantly differ between the combination-therapy and atezolizumab plus bevacizumab alone groups. Regarding complications associated with locoregional treatment in the combination-therapy group, transient grade-1 fever and grade-1 liver injury were observed after TACE (1/1 and 1/1, respectively) and RFA (4/9 and 9/9, respectively). A grade-one pneumothorax was observed after RFA for lung metastasis (1/1). Severe adverse events, such as bleeding that required blood transfusion or gastrointestinal perforation, did not occur in any patient.

**Figure 2:**
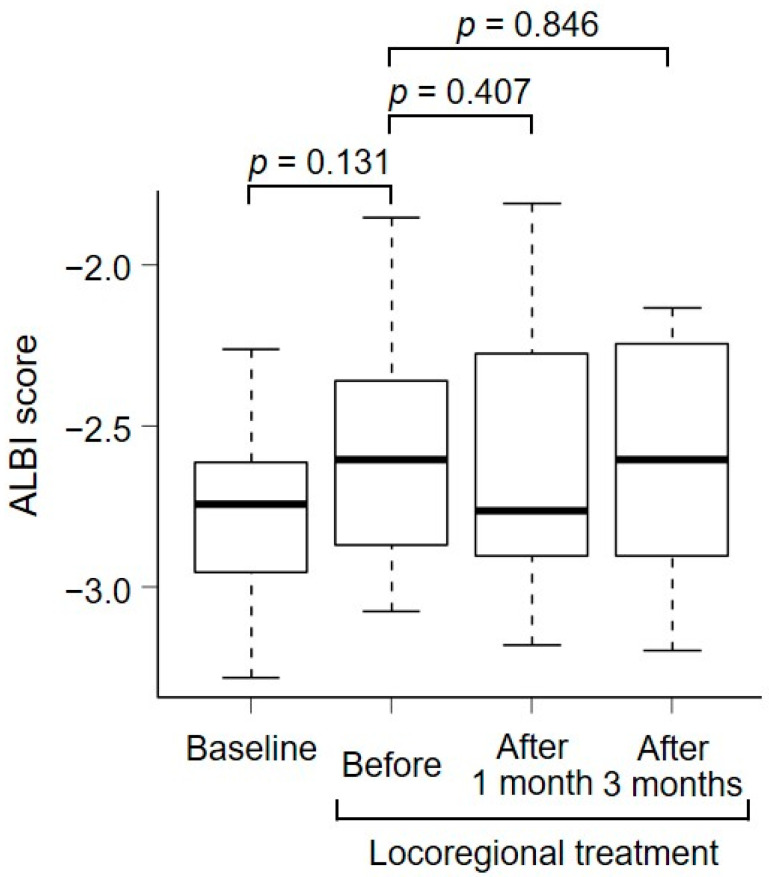
Changes in the ALBI scores in both pre- and post-locoregional treatment. The median ALBI scores at baseline (initiation of atezolizumab plus bevacizumab), before locoregional treatment, and at 1 month and 3 months after locoregional treatment were −2.743, −2.606, −2.763, and −2.604, respectively. ALBI, albumin–bilirubin.

Changes in the ALBI scores before and after locoregional treatment are shown in Figure 2. Among the ten patients in the combination-therapy group, the median ALBI scores at baseline (initiation of atezolizumab plus bevacizumab), before locoregional treatment, and at one month and three months after locoregional treatment were −2.743 (range, −3.283–2.263), −2.606 (range, −3.075–1.854), −2.763 (range, −3.181–1.810), and −2.604 (range, −3.198–2.134), respectively. No significant changes were observed after locoregional treatment. In the combination-therapy group, no patients experienced hepatic decompensation due to locoregional treatment or atezolizumab plus bevacizumab treatment. However, one patient in the atezolizumab plus bevacizumab alone group developed liver failure due to tumor rupture during atezolizumab plus bevacizumab treatment.

## 4. Discussion

Atezolizumab plus bevacizumab treatment results in an improved prognosis for patients with unresectable HCC. However, the treatment outcome remains unsatisfactory. During atezolizumab plus bevacizumab treatment, residual lesions were observed in some patients with tumor shrinkage, and a few drug-resistant lesions were observed in other patients. We analyzed the safety and feasibility of combining on-demand selective locoregional treatment for residual lesions after tumor shrinkage or for solitary or a few drug-resistant lesions with first-line atezolizumab plus bevacizumab for patients with unresectable HCC.

In this study, locoregional treatments were administered to determine CR or PD salvage status. The CR rate of the combination-therapy group was 50%; this was relatively higher than that of the atezolizumab plus bevacizumab alone group (10.5%), which was comparable to those reported by previous studies of atezolizumab plus bevacizumab treatment [7,8]. A durable response can be achieved with atezolizumab plus bevacizumab without reducing the hepatic reserve [30,31]; however, it is difficult to achieve a CR using atezolizumab plus bevacizumab alone [8,32,33]. This suggests that viable residual diseases may remain with a durable response to atezolizumab plus bevacizumab. Locoregional CR-oriented treatment should be considered for some patients with tumor shrinkage caused by atezolizumab plus bevacizumab. In contrast, executing locoregional salvage treatment for PD requires more careful consideration and judgment. Regarding solid tumors, the appearance of new tumors or the growth of drug-resistant lesions during systemic chemotherapy is considered to indicate PD, and the next treatment regimen should be planned accordingly. Nonetheless, distinctive locoregional treatment has been developed for localized HCC. Because of this progress, locoregional PD salvage treatments were selectively performed for cases meeting the following requirements: (1) the presence of solitary or few drug-resistant lesions; (2) low likelihood of tumor aggravation during the atezolizumab plus bevacizumab discontinuation period for locoregional treatment; and (3) locoregional treatment could be performed safely and minimally invasively without reducing hepatic reserve. Consequently, all patients successfully achieved PD salvage. The median discontinuation period of atezolizumab plus bevacizumab before and after locoregional treatment during this study was 49 days. During the discontinuation period of atezolizumab plus bevacizumab, tumors other than locoregional treatment-targeted lesions did not progress, and no new lesions appeared. Regarding the immune-checkpoint-inhibitor (ICI) responders, the binding of ICIs with CD8-positive T cells has been detected, and the clinical response was maintained for several weeks after the last administration [34,35]. Therefore, locoregional PD salvage treatment should probably only be directed toward a solitary or a few drug-resistant lesions that occur in response to atezolizumab plus bevacizumab. Extending the discontinuation period because of locoregional treatment-related complications should be avoided as much as possible, though several weeks of atezolizumab plus bevacizumab discontinuation for locoregional treatment may be acceptable.

The PFS (median, 7.4 months) and OS (median, 19.8 months) of the atezolizumab plus bevacizumab alone group were equivalent to that of previous clinical trials of atezolizumab plus bevacizumab [8,36]. In contrast, the PFS of patients who received combined selective locoregional treatment, such as surgical resection, RFA, TACE, and SBRT, with first-line atezolizumab plus bevacizumab administration (median not available) was significantly longer than that of the atezolizumab plus bevacizumab alone group. The OS of the combination-therapy group (median not available) was also longer than that of the atezolizumab plus bevacizumab alone group. The performance of locoregional treatment was independently associated with the prolongation of PFS and OS. The ORR of the combination-therapy group was higher than that of the atezolizumab plus bevacizumab alone group. On the other hand, no significant differences in the frequency of any grade or grade ≥ 3 adverse events were observed between the combination-therapy and atezolizumab plus bevacizumab alone groups. No patients in the combination-therapy group experienced hepatic decompensation due to locoregional treatment or atezolizumab plus bevacizumab treatment. Collectively, these factors could contribute to the observed favorable prognosis. Furthermore, locoregional treatment selectively targeted the remaining viable lesions after tumor shrinkage, which were smaller than the baseline tumor burden, or solitary or few drug-resistant lesions. The hepatic reserve was not reduced after locoregional treatment. This could also provide some clinical benefits because preserved liver function is an important prognostic factor in systemic therapy [37].

Recently, two phase-3 trials, namely the EMERALD-1 trial [38] and IMbrave050 trial [39], have demonstrated that combination immunotherapy after locoregional treatment prolonged PFS after TACE and recurrence-free survival after surgical resection or RFA, respectively. Thus, in the combination-therapy group, administration of atezolizumab plus bevacizumab could potentially yield an adjuvant therapy effect. Additionally, it is considered that RFA could enhance the immune response by inducing immunogenic cell death or by modulating the immune microenvironment. Because the antitumor effect is expected to be augmented following the activation of the immune system after the ablation of one tumor, leading to the recognition of other tumors in ICI-treated patients, this effect could also serve to prevent recurrence after achieving a CR or suppress tumor growth in the combination-therapy group.

The majority of patients in the combination-therapy group had BCLC Stage C disease in this study. However, most previous papers investigating combined systemic therapy with locoregional treatment have exclusively enrolled patients with BCLC Stage B disease [23,24]. Therefore, in line with recent reports [40], this study suggests the safety and feasibility of combining systemic therapy with selective locoregional treatment for patients with BCLC Stage C as well as BCLC Stage B, if selected appropriately.

Our study has some important limitations. Selection bias probably existed in the combination-therapy group. In the Kaplan–Meier analysis, the cumulative PFS and OS rates on day 224, which is the median day of the first locoregional treatment, were different between the combination-therapy and atezolizumab plus bevacizumab alone groups. These data suggest that the combination-therapy group may have included more atezolizumab plus bevacizumab-sensitive HCC cases compared to the atezolizumab plus bevacizumab alone group. The small sample size and retrospective single-center design may have also introduced some bias in patient selection. Furthermore, we collectively analyzed the safety and feasibility of four locoregional treatments—RFA, SBRT, surgical resection, and TACE—rather than assessing each treatment individually. Additionally, the lack of strict specifications regarding the intervals for radiological assessments may have affected the PFS values. To confirm the effectiveness of combination therapy during first-line atezolizumab plus bevacizumab, large-scale, randomized studies, including a group without locoregional treatment in such patients who underwent locoregional treatment in the present study and serving as a control for comparison with the locoregional treatment group, are required.

## 5. Conclusions

Patients with unresectable HCC who underwent on-demand selective locoregional treatment with first-line atezolizumab plus bevacizumab could safely achieve CR or PD salvage without a reduction in the hepatic reserve. Some clinical benefits were observed in the combination-therapy group. When performed in a minimally invasive manner, the combination of on-demand locoregional treatment targeting residual lesions after tumor shrinkage or solitary or few drug-resistant lesions with first-line atezolizumab plus bevacizumab could be feasible. Further investigation is required to validate our findings.

## Figures and Tables

**Table 1:** Comparison of characteristics of patients with or without combination therapy with atezolizumab plus bevacizumab.

		Atezolizumab Plus Bevacizumab		
Variable	All Patients (*n* = 29)	Combination Therapy (*n* = 10)	Atezo/Bev Alone (*n* = 19)	*p*-Value
Age, years	75 (46–85)	73 (62–85)	75 (46–82)	0.927
Sex, male/female	27/2	9/1	18/1	1.000
Etiology, HBV/HCV/NBNC	8/5/16	2/4/4	6/1/12	0.080
Child–Pugh score, 5/6/7	22/6/1	7/3/0	15/3/1	0.761
ALBI score	−2.615 (−3.283−1.772)	−2.743 (−3.283−2.263)	−2.518 (−3.011−1.772)	0.077
Modified ALBI grade, 1/2a/2b	16/8/5	8/1/1	8/7/4	0.185
Platelet count, 104/μL	18.4 (8.8–48.5)	15.3 (12.3–36.4)	18.7 (8.8–48.5)	0.506
Macrovascular invasion, +/−	2/27	1/9	1/18	1.000
Extrahepatic metastasis, +/−	17/12	6/4	11/8	1.000
BCLC Stage, B */C	10/19	3/7	7/12	1.000
NLR	3.275 (1.364–12.88)	4.812 (2.000–7.485)	2.907 (1.364–12.88)	0.085
Serum AFP, ng/mL	13 (2− > 10,000)	8 (2–2039)	57 (2− > 10,000)	0.160
Serum DCP, mAU/mL	175 (<30–220,629)	53 (<30 to 25,329)	699 (<30–220,629)	0.135

Continuous variables are shown as medians (ranges). * All patients with BCLC Stage B had HCC beyond the up-to-seven criteria. ALBI, albumin–bilirubin; BCLC, Barcelona Clinic Liver Cancer; NLR, neutrophil-to-lymphocyte ratio; AFP, α-fetoprotein; DCP, des-γ-carboxy prothrombin; +/−, with/without; HBV, hepatitis B virus; HCV, hepatitis C virus; NBNC, non-B, non-C hepatocellular carcinoma.

**Table 2:** Comparison of therapeutic efficacy between the combination-therapy group receiving locoregional treatment plus atezolizumab and bevacizumab, and the atezolizumab and bevacizumab alone group.

	Atezolizumab Plus Bevacizumab		
Variable	Combination Therapy (*n* = 10)	Atezo/Bev Alone (*n* = 19)	*p*-Value
Prior to the first combination therapy			
Best response, CR/PR/SD/PD	0/4/6/0	N.A.	
Objective response rate, %	40.0	N.A.	
Disease-control rate, %	100.0	N.A.	
Overall period			
Best response, CR/PR/SD/PD	5/3/2/0	2/2/10/5	
Objective response rate, %	80.0	21.1	**0.005**
Disease-control rate, %	100.0	73.7	0.134

A bold number indicates the *p*-value with statistical significance. CR, complete response; PR, partial response; SD, stable disease; PD, progressive disease; N.A., not applicable.

**Table 3:** Multivariate analysis of progression-free survival. Bold numbers indicate the *p*-values with statistical significance (*p* < 0.1 in the univariate analysis and *p* < 0.05 in the multivariate analysis).

	Univariate Analysis			Multivariate Analysis		
	HR	95% CI	*p*-Value	HR	95% CI	*p*-Value
Age, <75 vs. ≥75 years	1.117	0.430–2.901	0.820			
Sex, male vs. female	0.446	0.099–1.999	0.291			
Etiology, viral vs. nonviral	0.939	0.360–2.446	0.898			
Child-Pugh score, 5 vs. 6 or 7	1.547	0.439–5.446	0.497			
ALBI grade, 1 vs. 2	2.965	0.752–11.69	0.120			
BCLC stage, B vs. C	0.866	0.320–2.346	0.777			
NLR, <3 vs. ≥3	0.896	0.684–1.175	0.427			
Serum AFP, <200 vs. ≥200 ng/mL	1.620	0.597–4.401	0.344			
Serum DCP, <1000 vs. ≥1000 mAU/mL	2.273	0.870–5.937	**0.093**	2.320	0.864–6.231	0.095
Locoregional treatment, yes vs. no	0.183	0.051–0.658	**<0.001**	0.177	0.048–0.652	**0.004**

ALBI, albumin–bilirubin; BCLC, Barcelona Clinic Liver Cancer; NLR, neutrophil-to-lymphocyte ratio; AFP, α-fetoprotein; DCP, des-γ-carboxy prothrombin; CI, confidence interval; HR, hazard ratio.

**Table 4:** Multivariate analysis of overall survival.

	Univariate Analysis			Multivariate Analysis		
	HR	95% CI	*p*-Value	HR	95% CI	*p*-Value
Age, <75 vs. ≥75 years	1.325	0.479–3.666	0.588			
Sex, male vs. female	1.117	0.146–8.577	0.915			
Etiology, viral vs. nonviral	0.763	0.271–2.151	0.609			
Child-Pugh score, 5 vs. 6 or 7	1.465	0.383–5.609	0.577			
ALBI grade, 1 vs. 2	4.989	1.020–24.40	**0.047**	5.547	0.877–35.09	0.069
BCLC stage, B vs. C	0.958	0.324–2.832	0.938			
NLR, <3 vs. ≥3	0.418	0.147–1.189	0.102			
Serum AFP, <200 vs. ≥200 ng/mL	1.818	0.599–5.516	0.291			
Serum DCP, <1000 vs. ≥1000 mAU/mL	2.880	1.015–8.173	**0.047**	4.275	1.231–14.84	**0.022**
Locoregional treatment, yes vs. no	0.053	0.006–0.417	**<0.001**	0.027	0.003–0.285	**0.003**

Bold numbers indicate the *p*-values with statistical significance (*p* < 0.1 in the univariate analysis and *p* < 0.05 in the multivariate analysis). ALBI, albumin–bilirubin; BCLC, Barcelona Clinic Liver Cancer; NLR, neutrophil-to-lymphocyte ratio; AFP, α-fetoprotein; DCP, des-γ-carboxy prothrombin; CI, confidence interval; HR, hazard ratio.

**Table 5:** Adverse events experienced by patients in the combination-therapy group receiving locoregional treatment plus atezolizumab and bevacizumab, and the atezolizumab and bevacizumab alone group.

	Atezolizumab Plus Bevacizumab		
Variable	Combination Therapy (*n* = 10)	Atezo/Bev Alone (*n* = 19)	*p*-Value
Adverse events			
Yes/no	8/2	15/4	1.000
Grade ≥ 3	4	7	1.000
Liver injury	8	8	
Hypertension	5 (3)	5 (1)	
Proteinuria	2 (2)	5 (4)	
Adrenal cortical insufficiency	1	5	
Hypothyroidism	1	4	
Pneumonitis	1	0	
Tumor hemorrhage	1 (1)	2 (2)	
Subconjunctival hemorrhage	0	2	
Nosebleed	0	1	
Fever	4	1	
Skin rash	2	2	
Diarrhea	1	1	
Renal dysfunction	0	1 (1)	
Hyperamylasemia	0	1 (1)	
Fatigue	1	1	
Loss of appetite	0	1	
Stomatitis	1	0	
Thrombocytopenia	1	0	
Neutropenia	1	0	
Pneumothrax	1	0	

The numbers in parentheses indicate the number of patients classified as grade 3 or above. Atezo/bev, Atezolizumab plus bevacizumab.

## Data Availability

The data shown in this study are available from the corresponding author upon reasonable request.

## Supplementary Materials

The following supporting information can be downloaded at: https://www.mdpi.com/article/10.3390/curroncol31030117/s1, Figure S1: Schema of the combination of locoregional treatment with atezolizumab plus bevacizumab (atezo/bev); Figure S2: A representative case of sequential atezolizumab plus bevacizumab (atezo/bev) and CR-oriented locoregional treatment (Patient 10 in Table S1); Figure S3: A representative case of sequential atezolizumab plus bevacizumab (atezo/bev) and locoregional PD salvage treatment (Patient 6 in Table S1); Table S1: Summary of treatment information of the combination group patients.
