# Supplementary material for: Safety and Feasibility of Combining On-Demand Selective Locoregional Treatment with First-Line Atezolizumab Plus Bevacizumab for Patients with Unresectable Hepatocellular Carcinoma
Source: Curr Oncol. 2024 Mar 15;31(3):1543–55. doi: 10.3390/curroncol31030117 (PMC10969074; doi:10.3390/curroncol31030117)
Supplement: Supplementary file 1 [file curroncol-31-00117-s001.zip › 20240131_Supplementary_Material_ì┼ÉV.pdf]

Supplementary Material

Safety and feasibility of combining on-demand selective locoregional treatment with first-line atezolizumab plus bevacizumab for patients with unresectable hepatocellular carcinoma

Tasuku Nakabori*, Sena Higashi, Yutaro Abe, Kaori Mukai, Toshiki Ikawa, Koji Konishi, Noboru Maeda, Katsuyuki Nakanishi, Shinichiro Hasegawa, Hiroshi Wada, and Kazuyoshi Ohkawa

*** Correspondence:** Tasuku Nakabori, tasuku.nakabori@oici.jp

1 Supplementary Figures and Tables

1.1 Supplementary Figures

Supplementary Figure S1. Schema of the combination of locoregional treatment with atezolizumab plus bevacizumab (atezo/bev). Residual lesions after shrinkage by atezo/bev were treated by locoregional treatment to achieve a CR. This was called CR-oriented treatment in this paper (A). Solitary or few growing existing lesions (B, upper) or solitary new tumor (B, lower) during atezo/bev treatment were selectively treated by locoregional treatment. When intrahepatic lesions were controlled with atezo/bev, solitary or few extrahepatic lesions were also treated with locoregional treatment (C). (B) and (C) were called PD salvage treatment in this paper. Locoregional treatment was repeated if appropriate.

Supplementary Figure S2. A representative case of sequential atezolizumab plus bevacizumab (atezo/bev) and CR-oriented locoregional treatment (Patient 10 in Table S1). (A) A man in his 60s was initially diagnosed with unresectable hepatocellular carcinoma BCLC Stage B with a tumor burden of up-to-seven out (arrows; upper: arterial phase; lower: portal phase; left: segment (S) 8, 18.2 mm; middle: S5/8, 77.3 mm; right: S4, 26.1 mm). (B) After atezo/bev treatment was initiated, the S5/8 tumor shrunk and degenerated to a cystic lesion (arrow, middle; gadolinium-ethoxybenzyl diethylenetriamine pentaacetic acid-enhanced magnetic resonance imaging; upper: arterial phase; lower: hepatobiliary phase). The S8 tumor shrunk slightly, but its vascularity remained (arrow, left); furthermore, the size of the S4 tumor did not change, but its vascularity decreased (arrow, right). Atezo/bev provided a durable response, which was classified as a PR, but it did not achieve a CR. (C) After 14 courses of atezo/bev, RFA was performed for the S8 and S4 tumors. After RFA, atezo/bev was withdrawn, and the CR was maintained (upper: arterial phase; lower: portal phase).

BCLC, Barcelona Clinic Liver Cancer; CR, complete response; PR, partial response; RFA, radiofrequency ablation

Supplementary Figure S3. A representative case of sequential atezolizumab plus bevacizumab (atezo/bev) and locoregional PD salvage treatment (Patient 6 in Table S1). (A) A man in his 70s was diagnosed with a recurrence of hepatocellular carcinoma BCLC Stage C (four intrahepatic lesions and three lung metastases) (upper: lung metastases [arrows]; lower [arterial phase]: intrahepatic lesions [arrows]) six years after the initial diagnosis of hepatocellular carcinoma. He previously underwent left hepatic lobectomy, three transarterial chemoembolizations, and nine RFAs. (B) After

11 courses of atezo/bev, lung metastases shrunk (arrows), and vascularity of the intrahepatic lesions disappeared; however, a new intrahepatic lesion (diameter: 16.2 mm) with invasion into the inferior vena cava (Vv3) appeared (arrowhead) (upper: lung metastases [arrows]; lower [arterial phase]: intrahepatic lesions). Atezo/bev was discontinued, and SBRT was performed for the new intrahepatic tumor, including the Vv3 lesion. After SBRT, atezo alone was administered once, and systemic chemotherapy was discontinued again to perform RFA for four intrahepatic lesions as CR-oriented treatment. After RFA, atezo/bev was resumed. (C) After 24 courses of atezo/bev, lung metastasis in the left lower lobe (arrowhead) progressed; however, no new intrahepatic lesions occurred (upper: lung metastases; middle: portal phase). Atezo/bev was discontinued again, and RFA was performed for progressing lung metastasis (lower), followed by the continuation of atezo/bev.

BCLC, Barcelona Clinic Liver Cancer; CR, complete response; PD, progressive disease; PR, partial response; RFA, radiofrequency ablation; SBRT, stereotactic body radiotherapy

CR, complete response; PD, progressive disease

1.2 Supplementary Tables

Supplementary Table S1
