# Supplementary figures and images for: Safety and Feasibility of Combining On-Demand Selective Locoregional Treatment with First-Line Atezolizumab Plus Bevacizumab for Patients with Unresectable Hepatocellular Carcinoma
Source: Curr Oncol. 2024 Mar 15;31(3):1543–55. doi: 10.3390/curroncol31030117 (PMC10969074; doi:10.3390/curroncol31030117)

Figure S1

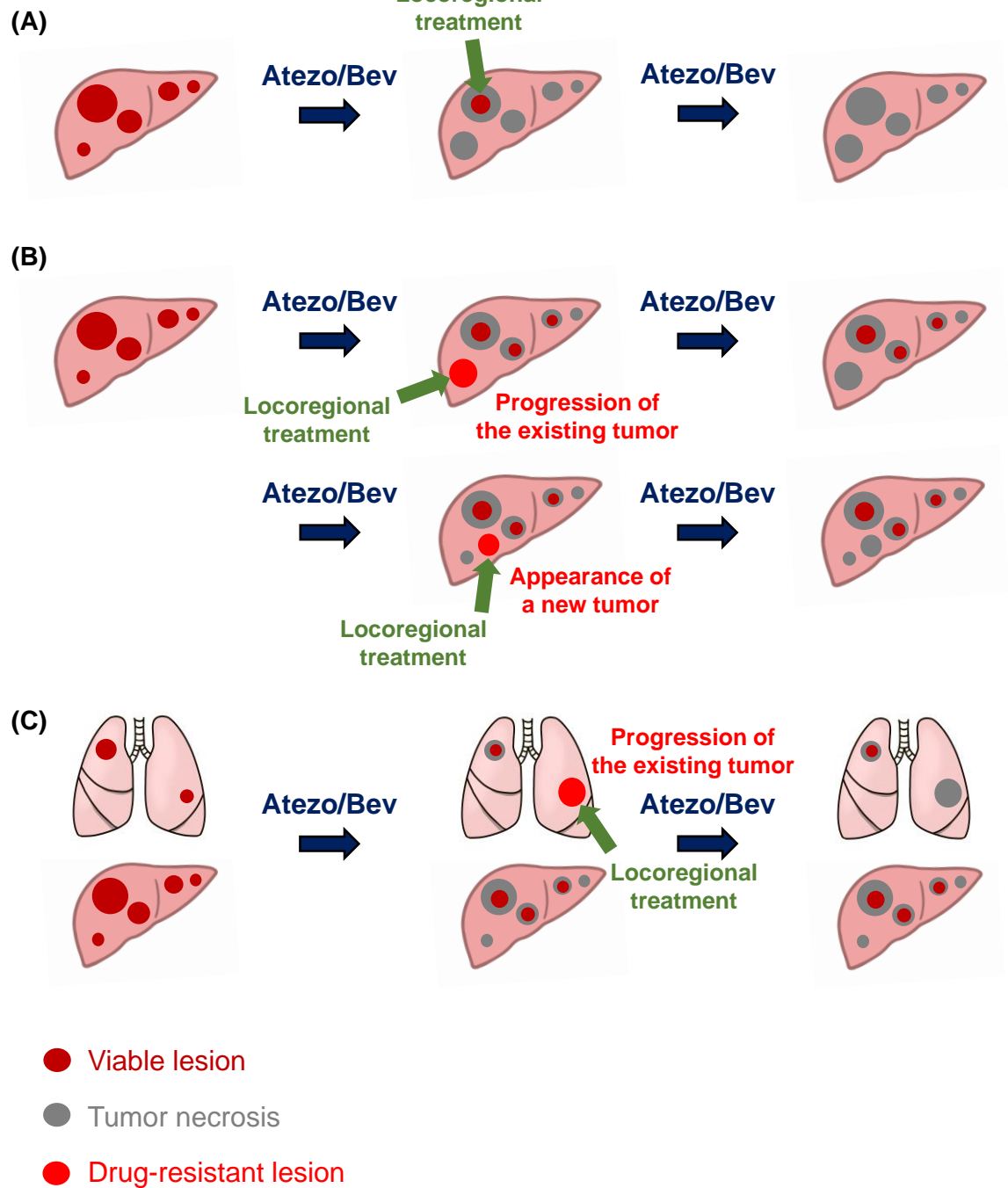

Supplement: Supplementary file 1 [file curroncol-31-00117-s001.zip › 20240131_Atezo_Bev_combination_FigS1_ì┼ÉV.pdf]

Figure S2

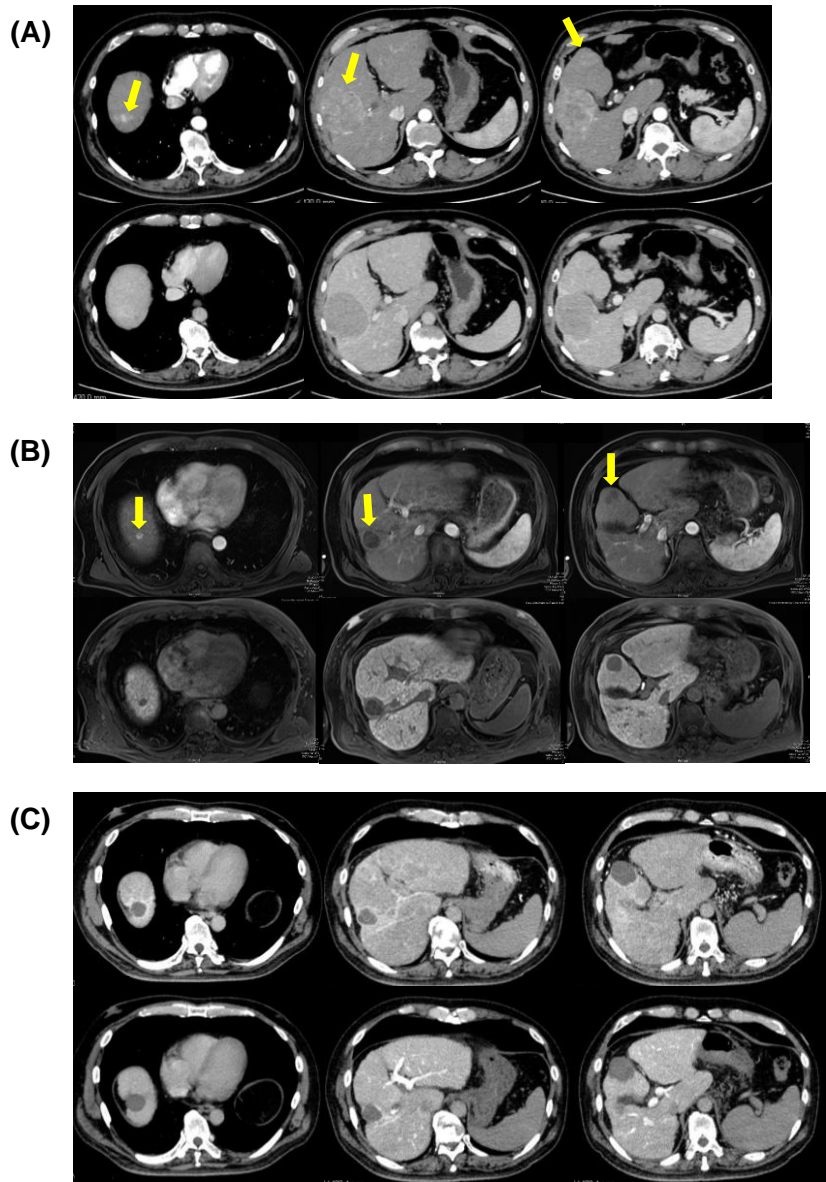

Supplement: Supplementary file 1 [file curroncol-31-00117-s001.zip › 20240131_Atezo_Bev_combination_FigS2_ì┼ÉV.pdf]

Figure S3

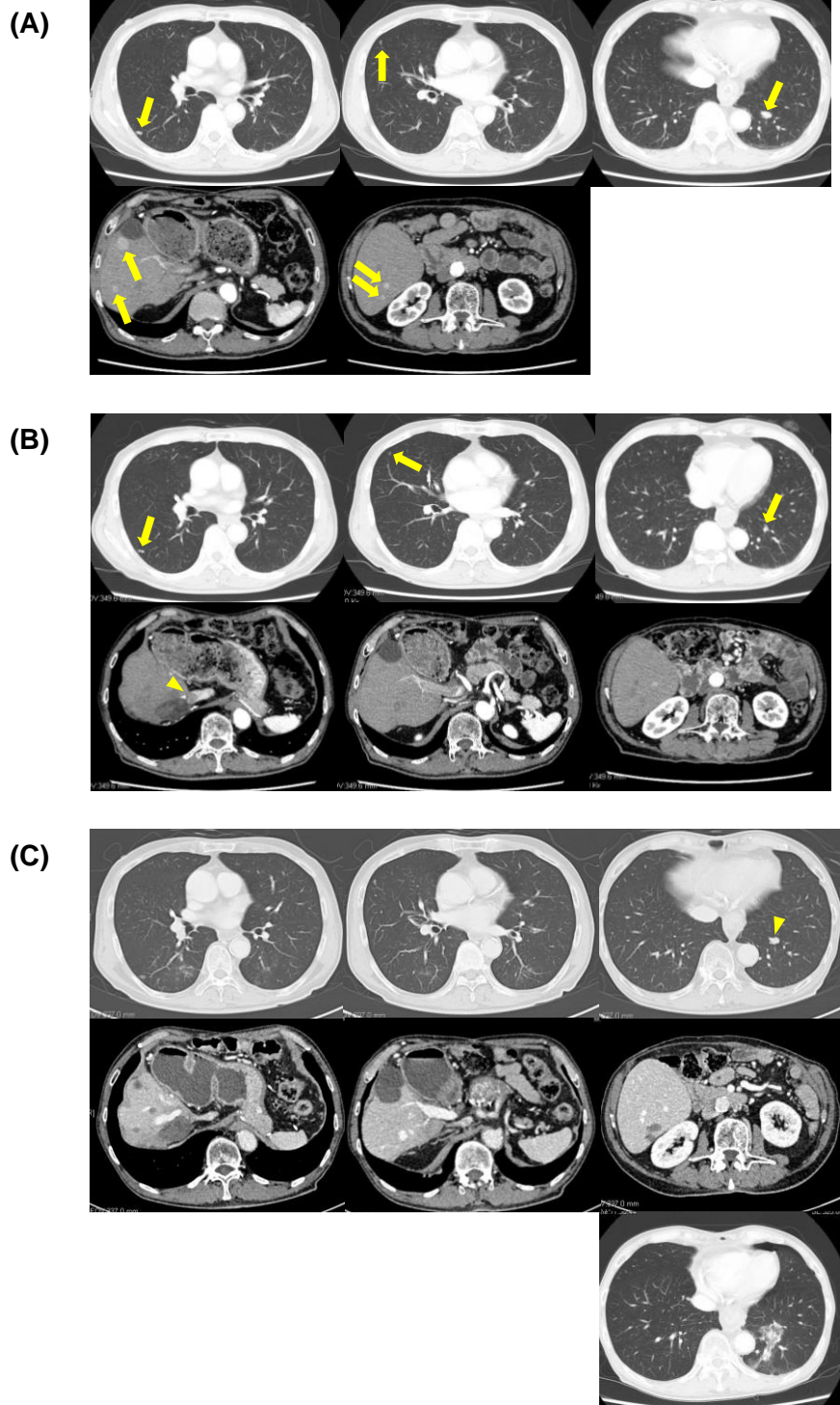

Supplement: Supplementary file 1 [file curroncol-31-00117-s001.zip › 20240131_Atezo_Bev_combination_FigS3_ì┼ÉV.pdf]
